# Top predator sea stars are the benthic equivalent to polar bears of the pelagic realm

**DOI:** 10.1073/pnas.2216701120

**Published:** 2022-12-27

**Authors:** Rémi Amiraux, David J. Yurkowski, Philippe Archambault, Marie Pierrejean, C. J. Mundy

**Affiliations:** ^a^Centre for Earth Observation Science, Clayton H. Riddell Faculty of Environment, Earth, and Resources, University of Manitoba, Winnipeg, MB R3T 2N2, Canada; ^b^Arctic and Aquatic Research Division, Fisheries and Oceans Canada, Winnipeg, MB R3T 2N6 Canada; ^c^Québec-Océan, ArcticNet, Sentinel North and Takuvik, Laval University, Québec, QC G1V 0A6, Canada

**Keywords:** Arctic Ocean, food web, trophic position, polar bear, sea star

## Abstract

The marine pelagic compartment spans numerous trophic levels and consists of numerous reticulate connections between species from primary producers to iconic apex predators, while the benthic compartment is perceived to be simpler in structure and comprised of only low trophic level species. Here, we challenge this paradigm by illustrating that the benthic compartment is home to a subweb of similar structure and complexity to that of the pelagic realm, including the benthic equivalent to iconic polar bears: megafaunal-predatory sea stars.

Food webs are a central concept in ecology that has provided considerable insight into ecosystem functioning, including patterns of energy and contaminant transfer as well as bottom-up or top-down trophic control of community structure (1, 2). At the base of the food web, primary producers support lower trophic levels that transfer the energy up to top predators (2). Generally, the trophic structure of the pelagic compartment of marine ecosystems is studied extensively (3), whereas the benthic compartment is studied much less often and is typically perceived to only consist of a truncated food chain with lower trophic level species (4). This paradigm has never been challenged although the benthic compartment has its own primary producers (5) (microphytobenthos, macroalgae) as well as the presence of at least 4 total trophic levels (6), suggesting a complete subweb with a complex structure. Here, we assessed an entire coastal Arctic marine food web to undertake the most comprehensive assessment of food chain length in both the pelagic and benthic compartments.

## Results and Discussion

To reveal that the benthic and pelagic compartments are two distinct, yet interconnected compartments, we analyzed 881 samples of benthic invertebrates belonging to 97 taxa and 9 phyla as well as 699 samples of pelagic fauna (invertebrates, demersal and pelagic fishes, seabirds, and marine mammals) belonging to 53 species from 12 taxonomic groups, in the Arctic marine waters around Southampton Island (Nunavut, Canada). The trophic structure of this system increased to an average trophic position (TP) of 5.7 which is slightly higher than that in some areas across the pan Arctic (3, 7).

**Fig. 1.**
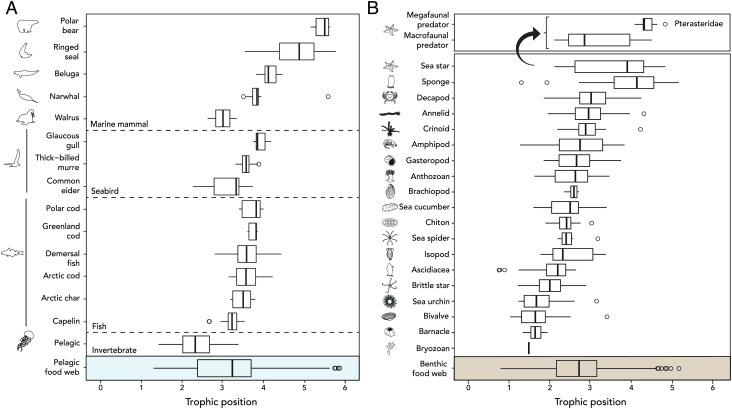
TP of organisms collected around Southampton Island. TP of organisms belonging from the (*A*) pelagic subweb and (*B*) benthic subweb.

In order to consider the benthic compartment as hosting a complex and comprehensive subweb of the marine food web rather than as a reservoir of lower trophic level species for the pelagic subweb, it is necessary for the latter to possess several trophic levels including the presence of its own top predators. A similar average TP was found between the benthic and pelagic compartments (2.9 ± 0.7 and 3.2 ± 0.9, respectively) associated with a similar range of TPs ([1.1 to 4.8] and [1.6 to 5.7], respectively) (Fig. 1), indicating a comparable structure and complexity between these two compartments. Our study, taking place in the coastal Arctic region of Southampton Island, Nunavut, concludes that shelf benthic ecosystems can have a more complex trophic structure than previously expected with up to nearly five trophic levels comparable to that of the deep ocean and pelagic compartment (6). This complexity of the benthic ecosystem is also emphasized by the high variability in TPs of its taxa, which highlights the important feeding plasticity of the species that compose them. For instance, sea stars as a general grouping had the greatest range of TP with low values derived from *Leptasterias (Hexasterias) polaris* and *Leptasterias groenlandica* (TP of 2.1 ± 0.0 and 2.4 ± 0.2, respectively) that are known to feed primarily on low trophic level taxa such as bivalves and molluscs (8). The highest trophic levels occurred in Pterasteridae—*Diplopteraster multipes, Pteraster militaris,* and an unidentified *Asteroidea* (TP of 4.2 ± 0.6, 4.7 ± 0.1, and 5.0, respectively), which are all known to rely on megafaunal prey of higher trophic level (e.g., Porifera, Cnidaria) (9, 10) (Fig. 1*B*). Because of their diet on high trophic level organisms and their lack of predators—likely due to their evolved highly efficient defense mechanisms (11)—these megafaunal predatory sea stars occupy the highest TP in the benthos (Fig. 1*B*) and are therefore the apex predator of the Arctic benthic environment. As such, sea stars should be considered as the benthic counterpart of the pelagic apex predator: the polar bear. The apex predator position of sea stars is also corroborated by their feeding type such as polar bears that are top predators and opportunistic scavengers. Indeed, recent studies suggest that beached large whale carcasses enhance polar bear survival during periods of low sea ice (12), while by inhabiting the benthic compartment that is the receiving area for all pelagic materials produced, sea stars are also scavengers of the carcasses of upper pelagic predators (13). This ability of predators to switch between hunting and scavenging (facultative scavenging) carries both short-term survival and long-term fitness advantages, enabling them to be apex predators (14). The persistence of apex predators showing this flexibility is probably a sign of a bottom-up-directed food web. Although this finding seems contradictory to Boyce's highlighting that Arctic food webs are top-down controlled (15), we suggest that Arctic food webs may actually switch seasonally from bottom-up to top-down control because of its pulsative production period. Finally, by virtue of their global distribution (16) and apex predator status, we confirm the role of sea stars as a ubiquitous key species in the oceanic realm (17, 18).

**Fig. 2.**
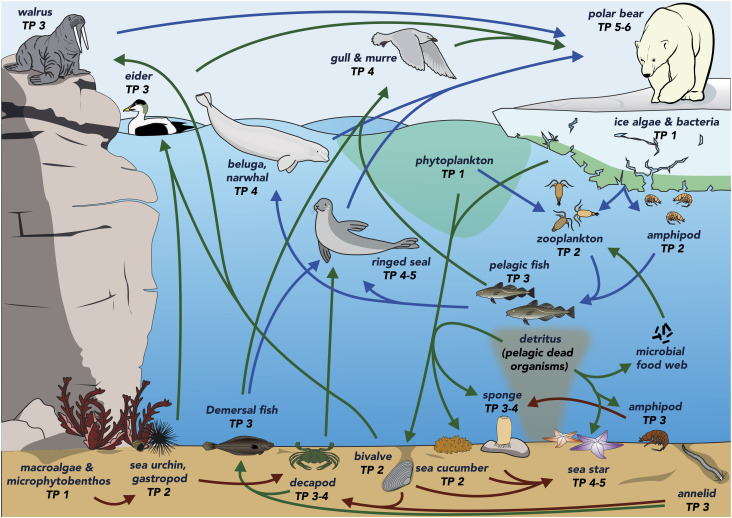
Schematic representation of the subarctic marine food web. Arrows indicate trophic interaction between species within the pelagic subweb (blue), the benthic subweb (brown), or between different subwebs (green).

The misconception that the benthic compartment only hosts lower trophic level species may have been related to the logistical difficulties of sampling this environment, the relatively small size of its organisms, the lack of charismatic top predator species in comparison to those of the pelagic compartment (e.g., polar bear, orca), and the numerous trophic interactions between benthic and pelagic species, perceived to arise from their belonging to the same subweb (Fig. 2). However, there are many examples of species interactions between different environments (e.g., marine-terrestrial, ref. 19; limnic-terrestrial, ref. 20), known as habitat coupling, and these seem essential to the functioning of the Earth (21). A more specific example within marine environments is the supply of nutrients via the marine microbial loop to pelagic primary production, which supports marine food web (22). These habitat couplings result from mobile, opportunistic species (23), which in our study include, but are not limited to, the walrus feeding on benthic bivalves, the shorthorn sculpin (demersal fish) feeding on both benthic and pelagic compartments, and the common eider (seabird) foraging on benthic organisms (Fig. 2). Although the strength of these interconnections between the benthic and pelagic subwebs remains understudied and was not able to be quantified in this study, they are numerous (Fig. 2) and thus, it is likely that the recognized top-down control of sea stars (17) in the benthic subweb also impacts the functioning and structure of the pelagic subweb, along with its apex predator. For example, increasing top-down control of sea stars on bivalves will alter walrus energy acquisition and space use, which in turn will affect polar bears. This assumption is supported by the numerous studies highlighting the worldwide deleterious effect of bottom trawling of benthic species on the pelagic subweb structure and complexity (24) and suggests that marine conservation initiatives should not overlook the benthic compartment.

We conclude that benthic compartments host fully fledged subwebs with megafaunal predatory sea stars as the benthic apex predator in the coastal regions of the Arctic and likely in other oceanic regions. Furthermore, since food webs are all interconnected, the role of sea stars in shaping the benthic subweb can cascade up to the pelagic subweb and require special attention to accurately quantify marine ecosystem structure and function.

## Materials and Methods

Nitrogen stable isotope analyses were performed after lipid extraction following (25) on whole organisms or soft parts for invertebrates; on muscles for fish, seals, and whales; and fur for polar bears. We analyzed red blood cells from seabirds, except for common eider whose plasma was analyzed to reflect their short-term diet. TP was calculated for each species using a one-source TP model to determine food chain length of each habitat compartment. A diet–tissue discrimination factor (Δ^15^N) of 3.4‰ was used for all species except narwhal, beluga, and ringed seals, which are primarily piscivorous, and 2.4‰ was applied as a Δ^15^N, with Arctic cod and capelin mean TP of 3.4 employed as a baseline following a scaled TP framework (3).

## Supplementary Material

Appendix 01 (PDF)

Dataset S01 (XLSX)

Dataset S02 (CSV)

## Data Availability

All study data are included in the *SI Appendix*.
