# Supplementary material for: Top predator sea stars are the benthic equivalent to polar bears of the pelagic realm
Source: Proc Natl Acad Sci U S A. 2022 Dec 27;120(1):e2216701120. doi: 10.1073/pnas.2216701120 (PMC9910421; doi:10.1073/pnas.2216701120)
Supplement: Supplementary file 1 — Appendix 01 (PDF) [file pnas.2216701120.sapp.pdf]

PNAS

Supporting Information for

Top predator seastars are the benthic equivalent to polar bears of the pelagic realm

Rémi Amiraux, David J. Yurkowski, Philippe Archambault, Marie Pierrejean, C.J. Mundy

Rémi Amiraux
Email: remi.amiraux@umanitoba.ca

This PDF file includes:

Supporting text
SI References

Other supporting materials for this manuscript include the following:

Datasets S1 to S2

Supporting Information Text

Extended material and methods

Sample collection. Benthic and pelagic invertebrate, demersal and pelagic fish, seabird and marine mammal samples were collected in summer 2016, 2018, and 2019 in the marine waters around Southampton Island, Nunavut, Canada. In the present paper, benthic subweb refers to the benthic invertebrates while the pelagic food web encompasses pelagic invertebrates, demersal and pelagic fishes, seabirds and marine mammals. Benthic and pelagic invertebrates and fishes were collected using a Campelen 1200 research trawl on the *MV Nuliajuk* in 2016 and using benthic and pelagic nets and trawls as well as a Ponar grab and 30-cm box corer on the *RV William Kennedy* in 2018 and 2019 as part of the Southampton Island Marine Ecosystem Project (SIMEP). Pelagic zooplankton were collected with an obliquely towed bongo net (500-μm mesh). For larger zooplankton and pelagic fish, a 3-m mid-water trawl (0.5-cm cod-end mesh) was towed at between 2-3 knots for 15 minutes targeting the subsurface chlorophyll maximum depth (SCM). A 3-m benthic beam trawl (0.5-cm cod-end mesh) was towed at 2-3 knots for 15 minutes on the bottom. Samples were sorted into main groups of zooplankton and identified to the lowest possible taxon level for benthic invertebrates and fishes in the field and were then frozen at –20°C for later food web analysis.

Benthic invertebrates consisted of 881 whole organism, muscle or soft part samples from 97 taxa belonging to 9 phyla as follows: Annelida, Arthropoda, Brachiopoda, Bryozoa, Chordata, Cnidaria, Echinodermata, Mollusca, Porifera. Pelagic invertebrates consisted of 303 whole organism samples belonging to 20 species and 8 taxonomic groups as follows: amphipod, chaetognath, copepod, ctenophore, hydrozoan, krill and mysid, pteropod, and squid.

Fish sample consisted in total of 171 muscle samples from 20 demersal (i.e. relying on both pelagic and benthic resources; *1*) fish species and 83 muscle samples from 5 pelagic species. Fish species included Arctic cod (*Boreogadus saida*), Polar cod (*Arctogadus glacialis*), Fourline snakeblenny (*Eumesogrammus praecisus*), Arctic staghorn sculpin (*Gymnocanthus tricuspis*), Arctic alligatorfish (*Aspidophoroides olrikii*), Daubed shanny (*Leptoclinus maculatus*), Fish doctor (*Gymnelus viridis*), Twohorn sculpin (*Icelus bicornis*), Spatulate sculpin (*Icelus spatula*), Atlantic poacher (*Leptagonus decagonus*), Snailfish (*Liparis* sp), Slender eelblenny (*Lumpenus fabricii*), Capelin (*Mallotus villosus*), Arctic sculpin (*Myoxocephalus scorpioides*), Shorthorn sculpin (*Myoxocephalus scorpius*), Greenland cod (*Gadus ogac*), Banded gunnel (*Pholis fasciata*), Arctic shanny (*Stichaeus punctatus*), Eelpouts (*Zoarcidae* sp), Moustache sculpin (*Triglops murrayi*), and Ribbed sculpin (*Triglops pingelii*). Arctic char (*Salvelinus alpinus*) muscle samples were collected opportunistically as part of Inuit subsistence harvests and in association with ongoing community-based monitoring programs with Fisheries and Oceans Canada based in Naujaat, Nunavut.

Marine mammal collections consisted of 72 muscle samples belonging to Atlantic walrus (*Odobenus rosmarus rosmarus*), ringed seal (*Phoca hispida*), narwhal (*Monodon monoceros*) and beluga (*Delphinapterus leucas*) that were collected in June to August by Inuit hunters from Naujaat and Coral Harbour, Nunavut as part of their subsistence harvests and ongoing community-based monitoring programs in collaboration with Fisheries and Oceans Canada. Polar bear (*Ursus maritimus*) collection comes from Koehler, Kardynal and Hobson (*2*) and consisted of fur collected in 2016 and 2017 in Foxe Basin.

Samples from three bird species were collected (n = 62 samples) by way of long-term monitoring programs coordinated by Environment and Climate Change Canada in East Bay and at the Coats Island murre west colony studied by McGill University, respectively. These samples consisted of blood from Glaucous gulls (*Larus hyperboreus*) and Thick-billed murres (*Uria lomvia*) and plasma from Common eiders (*Somateria mollissima*). For avian blood components, plasma has a faster turnover rate (e.g., days), while red blood cells have a longer turnover rate (e.g., weeks; *3-5*). Plasma was analyzed for the Common Eiders as they were pre-incubating at the time of their capture, which took place shortly after arrival in East Bay Island (see Smith, Yurkowski, Parkinson, Fort, Hennin, Gilchrist, Hobson, Mallory, Danielsen and Garbus (*6*) for more details), allowing us to quantify short-term diet after their arrival. In contrast, murres and gulls were sampled during chick-rearing, over a month after arrival at the site, and, thus, red blood cells should reflect local sources. All tissue samples were frozen at –20°C and shipped to the Freshwater Institute in Winnipeg, Manitoba before processing.

Nitrogen stable isotope analysis. Frozen samples were lyophilized for 48 hours at -50°C and then crushed into a fine powder using a mortar and pestle. Due to the ubiquitous presence of lipids in Arctic consumers including invertebrates (*7*), fish liver and muscle (*8*), and marine mammal muscle (*9*), lipids were extracted with 2:1 chloroform:methanol solvent using a modified version of the Bligh and Dyer (*10*) method for another paper investigating **δ^{13}C** variability. Although chemical lipid extraction can slightly affect δ^{15}N values in some cases, these influences vary in magnitude, but are generally small (<0.5‰;*9*) and much less than the diet-tissue discrimination factors applied below (3.4‰ and 2.4‰), so this would not lead to trophic misinterpretation. In addition, although sea stars are rich in carbonates that are enriched in ^{13}C, the carbonates were not extracted by acidification which might have slightly influenced δ^{15}N values, and instead we used correction factors using a carbonate proxy value of 1.1 similar to those reported by Kazanidis, Bourgeois and Witte (*11*). Stable isotope analysis was performed at the Chemical Tracers Laboratory, Great Lakes Institute for Environmental Research, at the University of Windsor using a Delta V Advantage Mass spectrometer (Thermo Finnigan, San Jose, CA, USA) coupled to a Costech 4010 Elemental

Combustion system (Costech, Valencia, CA, USA) and a ConFlo IV gas interface. For **δ^{15}N** analysis, subsamples of 400-600 µg of tissue were weighed into tin capsules. Stable isotope ratios are expressed in per mil (‰) in standard delta (**δ**) notation relative to the international standards atmospheric N_2 for nitrogen, using the following equation:

$$\delta X = [(\frac{\text{Rsample}}{\text{Rstandard}}) - 1] \times 10^3 \quad \text{(Eq. 1)}$$

Where X is ^{15}N and R equals $^{15}N/^{14}N$. Instrumentation accuracy checked throughout the period of time that these samples were analyzed, was based on NIST standards 8573, 8547 and 8574 (n=50 for all). The mean difference from the certified values were 0.09, 0.14, 0.06‰. Precision, assessed by the standard deviation of replicate analyses of four standards (NIST1577c, internal lab standard, tilapia muscle), USGS 40 and Urea (n=22 for all), was ≤0.25‰ for all the standards. We converted the stable nitrogen isotopes of Polar bears fur to those of their diet using the discrimination factors (*12*).

Trophic position (TP) was calculated for each species/taxonomic group using a one-source TP model (*13*; Eq. 2) to determine food chain length of each habitat compartment.

$$TP_{consumer} = TP_{baseline} + \frac{\delta^{15}N_{consumer} - \delta^{15}N_{baseline}}{\Delta 15N} \quad \text{(Eq. 2)}$$

The δ^{15}N of primary producers can sometimes vary between benthic and pelagic systems (*13*) however, in this study system, the pelagic-feeding copepod δ^{15}N value (*Calanus hyperboreus*; 9.5 ± 0.4‰) was comparable to a more-benthic associated grazer (i.e., the sea urchin *Strongylocentrotus droebachiensis*, Paar, Lebreton, Graeve, Greenacre, Asmus and Asmus (*14*); δ^{15}N of 8.7 ± 1.6‰) and filter-feeding species (i.e., bivalve, δ^{15}N of 8.5 ± 1.7‰). Therefore, the following one-source trophic position model with copepod as the baseline (TP=2) was applied to determine the TP of all taxa. A diet–tissue discrimination factor (Δ^{15}N) of 3.4‰ (*13*) was used for all species. Exceptions were narwhal, beluga and ringed seals, which are primarily piscivorous and 2.4‰ was applied as a Δ^{15}N (*15*), with Arctic cod and capelin mean TP of 3.4 employed as a baseline following a scaled trophic position framework (*16*). Dataset S1 (separate file). Mean Nitrogen stable isotope and trophic position of organisms in the subarctic marine food web around Southampton Island at the specie level.

Dataset S1 (separate file). Mean Nitrogen stable isotope and trophic position of organisms in the subarctic marine food web around Southampton Island at the specie level.

Dataset S2 (separate file). Nitrogen stable isotope and trophic position of organisms in the subarctic marine food web around Southampton Island at the specie level.

SI References

1. J. J. Landry *et al.*, Feeding ecology of a common benthic fish, shorthorn sculpin (Myoxocephalus scorpius) in the high arctic. *Polar Biology* **41**, 2091-2102 (2018).https://doi.org/10.1007/s00300-018-2348-8.
2. G. Koehler, K. J. Kardynal, K. A. Hobson, Geographical assignment of polar bears using multi-element isoscapes. *Scientific reports* **9**, 9390 (2019).https://doi.org/10.1038/s41598-019-45874-w.
3. V. Barquete, V. Strauss, P. G. Ryan, Stable isotope turnover in blood and claws: A case study in captive African Penguins. *Journal of Experimental Marine*

Biology and Ecology **448**, 121-127 (2013).https://doi.org/10.1016/j.jembe.2013.06.021.
4. S. Hahn, B. J. Hoye, H. Korthals, M. Klaassen, From food to offspring down: tissue-specific discrimination and turn-over of stable isotopes in herbivorous waterbirds and other avian foraging guilds. *PloS one* **7**, e30242 (2012).https://doi.org/10.1371/journal.pone.0030242.
5. K. A. Hobson, R. G. Clark, Assessing avian diets using stable isotopes I: turnover of 13C in tissues. *The Condor* **94**, 181-188 (1992).https://doi.org/10.2307/1368807.
6. R. A. Smith *et al.*, Environmental and life-history factors influence inter-colony multidimensional niche metrics of a breeding Arctic marine bird. *Science of The Total Environment* **796**, 148935 (2021).https://doi.org/10.1016/j.scitotenv.2021.148935.
7. A. B. Imbs, E. V. Ermolenko, V. P. Grigorchuk, T. V. Sikorskaya, P. V. Velansky, Current Progress in Lipidomics of Marine Invertebrates. *Marine drugs* **19**, 660 (2021)
8. D. M. Post *et al.*, Getting to the fat of the matter: models, methods and assumptions for dealing with lipids in stable isotope analyses. *Oecologia* **152**, 179-189 (2007).https://doi.org/10.1007/s00442-006-0630-x.
9. D. J. Yurkowski, N. E. Hussey, C. Semeniuk, S. H. Ferguson, A. T. Fisk, Effects of lipid extraction and the utility of lipid normalization models on δ13C and δ15N values in Arctic marine mammal tissues. *Polar Biology* **38**, 131-143 (2015).https://doi.org/10.1007/s00300-014-1571-1.
10. E. G. Bligh, W. J. Dyer, A rapid method of total lipid extraction and purification. *Canadian Journal of Biochemistry and Physiology* **37**, 911-917 (1959).https://doi.org/10.1139/o59-099.
11. G. Kazanidis, S. Bourgeois, U. F. Witte, On the effects of acid pre-treatment on the elemental and isotopic composition of lightly-and heavily-calcified marine invertebrates. *Ocean Science Journal* **54**, 257-270 (2019).https://doi.org/10.1007/s12601-019-0014-x.
12. V. L'Hérault, N. Lecomte, M. H. Truchon, D. Berteaux, Discrimination factors of carbon and nitrogen stable isotopes from diet to hair in captive large Arctic carnivores of conservation concern. *Rapid Communications in Mass Spectrometry* **32**, 1773-1780 (2018).https://doi.org/10.1002/rcm.8239.
13. D. M. Post, Using stable isotopes to estimate trophic position: models, methods, and assumptions. *Ecology* **83**, 703-718 (2002).https://doi.org/10.1890/0012-9658(2002)083[0703:USITET]2.0.CO;2.
14. M. Paar *et al.*, Food sources of macrozoobenthos in an Arctic kelp belt: trophic relationships revealed by stable isotope and fatty acid analyses. *Marine Ecology Progress Series* **615**, 31-49 (2019).https://doi.org/10.3354/meps12923.
15. K. A. Hobson, D. M. Schell, D. Renouf, E. Noseworthy, Stable carbon and nitrogen isotopic fractionation between diet and tissues of captive seals: implications for dietary reconstructions involving marine mammals. *Canadian Journal of Fisheries and Aquatic Sciences* **53**, 528-533 (1996).https://doi.org/10.1139/f95-209.

16. N. E. Hussey *et al.*, Rescaling the trophic structure of marine food webs. *Ecology letters* **17**, 239-250 (2014).https://doi.org/10.1111/ele.12226.
